# Analysis of the effects of evaporative cooling on the evaporation of liquid droplets using a combined field approach

**DOI:** 10.1038/srep08614

**Published:** 2015-02-27

**Authors:** Xuefeng Xu, Liran Ma

**Affiliations:** 1 School of Technology, Beijing Forestry University, Beijing 100083, China; 2 State Key Laboratory of Tribology, Tsinghua University, Beijing 100084, China

## Abstract

During liquid evaporation, the equations for the vapor concentration in the atmosphere and for the temperature in the liquid are coupled and must be solved in an iterative manner. In the present paper, a combined field approach which unifies the coupled fields into one single hybrid field and thus makes the iteration unnecessary is proposed. By using this approach, the influences of the evaporative cooling on the evaporation of pinned sessile droplets are investigated, and its predictions are found in good agreement with the previous theoretical and experimental results. A dimensionless number *Ec* which can evaluate the strength of the evaporative cooling is then introduced, and the results show that both the evaporation flux along the droplet surface and the total evaporation rate of the droplet decrease as the evaporative cooling number *Ec* increases. For drying droplets, there exists a critical value *Ec*_Crit_ below which the evaporative cooling effect can be neglected and above which the significance of the effect increases dramatically. The present work may also have more general applications to coupled field problems in which all the fields have the same governing equation.

The evaporation of liquid droplets is not only a common phenomenon in daily life, but also a fundamental process that impacts a wide range of industrial and scientific applications. For example, a sessile droplet often leaves the solute particles on the substrate on which it rested, resulting in different patterns of deposition upon drying^1^,^2^,^3^,^4^,^5^,^6^,^7^,^8^. This phenomenon has been used as the basis of many applications including DNA-RNA mapping^9^,^10^, ink-jet printing of functional materials^11^,^12^,^13^, and fabrication of colloidal photonic crystals^14^. In these applications, controlling the distribution of the particle deposition after the liquid has dried is vital. A better understanding of the evaporation process of the sessile droplets could be very helpful to such a goal.

Due to its crucial role in applications, droplet evaporation has attracted extensive attention over the past few decades^15^,^16^,^17^,^18^,^19^,^20^,^21^,^22^,^23^,^24^. For steady-state diffusion-controlled evaporation, the concentration of the liquid vapor above the sessile droplet satisfies the Laplace's equation. By using the known solution of an equivalent electrostatic problem^25^, the exact solution for the evaporation flux along the surface of pinned sessile droplets was derived by Picknett and Bexon^26^, Deegan et al.^2^, and Popov.^15^ A simple approximate expression for the evaporation flux along the droplet surface was then obtained numerically by Hu and Larson^27^ and found consistent well with the exact analytic expression and with the previous literature data.

In the above mentioned studies, however, the effect of the evaporative cooling on the vapor concentration and on the evaporation rate at the droplet surface has not been considered. All the above works are based on the assumptions that the atmosphere just above the droplet surface is saturated with vapor and that the saturation concentration of vapor is a constant along the surface. Actually, the evaporation will lower the temperature of the liquid at the droplet surface, which can in turn alter the saturation concentration of vapor there^18^,^28^,^29^,^30^,^31^,^32^,^33^,^34^,^35^,^36^,^37^. This means that the vapor concentration along the free surface of the droplets is often not uniform, different to what is assumed in the above literatures. Neglecting this evaporative cooling effect may introduce considerable discrepancy in predicting the evaporation rate of the drying droplets, especially when a large temperature reduction is induced at the droplet surface^30^,^34^,^38^,^39^,^40^,^41^.

By allowing the saturation concentration of vapor just above the droplet surface to be a function of temperature rather than simply a constant, the theoretical model of the droplet evaporation was generalized to include the effect of the evaporative cooling by Dunn et al.^28^,^29^ Sefiane et al.^30^, and Saada et al.^31^ The coupled problem for the vapor concentration and the temperature was solved numerically and the results showed that the thermal conductivities of the liquid and the substrate, the thickness of the substrate, and the atmospheric pressure have significant effects on the evaporation rate of sessile droplets. When taking into account the thermal effects resulting from evaporative cooling, Sefiane and Bennacer^34^,^35^,^36^ developed a theoretical expression for the evaporation rate of sessile droplets. A dimensionless number ***SB*** is also introduced to identify the threshold for the transition from an isothermal case to a nonisothermal one. Their theory is supported by a very wide range of experimental measurements.

Despite the theoretical and experimental researches of Dunn et al., Sefiane et al., Saada et al., and Sefiane and Bennacer, a complete theory for the evaporative cooling effect in a pinned sessile droplet is still lacking. A numerical quantity that can evaluate the strength of the evaporative cooling is also needed, for example, to investigate the dependence of the evaporation on the thermal properties of the liquid and on the atmospheric pressure. Because the problem of the vapor concentration is coupled with the problem of the temperature, the iteration between these two coupled physics fields has to be carried out in the computation procedure. Finding an efficient way to compute directly the vapor concentration field and the temperature field without iteration can benefit not only the evaporation of the sessile droplets but also other coupled-field problems.

In the present paper, a combined field approach is first introduced for the evaporation of the liquid droplets. This approach can unify the coupled physics fields into one single hybrid field and thus make the iteration unnecessary. By using the approach, a dimensionless number which can indicate the strength of the evaporative cooling during the liquid evaporation is derived, and then, the influences of the evaporative cooling on the evaporation process of the sessile droplets are discussed. The theoretical predictions are found in good agreement with the previous theoretical and experimental results. The present study may contribute to the body of knowledge concerning the droplet evaporation and thus may be useful to control the flow and the deposition of drying droplets.

## Results and Discussion

### Mathematical Model

**Figure 1:**
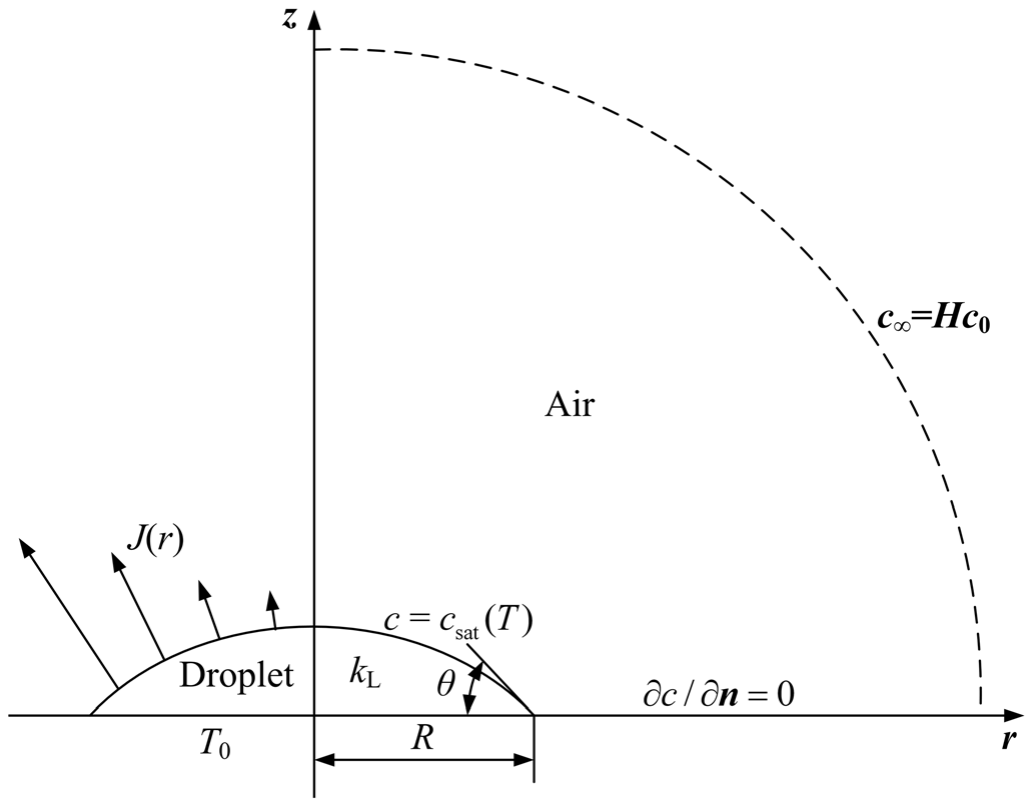
A spherical-cap evaporating liquid droplet on a flat substrate in a cylindrical coordinate system with radial coordinate *r* and axial coordinate *z*.

Here, we focus our study on the effects of the cooling at droplet surface on the evaporation of the droplet, and leave out of account the influences of the substrate. In the present model, we consider a small, pinned, and slowly evaporating liquid droplet with contact angle of *θ* and contact line radius of *R* resting on a flat isothermal substrate with constant temperature *T*_0_ which is equal to the room temperature. For the small and slowly evaporating droplet, the droplet shape can be regarded as a spherical cap due to its small Bond number and capillary number, and the height of the droplet can be expressed as 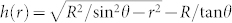
2,6,18,23,27,28. Due to the axisymmetric configuration, a cylindrical coordinate system (*r*, *z*) is chosen (Figure 1).

During the evaporation of small and slowly evaporating droplets, the time required for the vapor concentration to adjust to the change in the droplet shape is typically much smaller compared to the droplet evaporation time. Hence, the vapor diffusion in the atmosphere can be considered as quasi-steady, and the vapor concentration *c* in the atmosphere satisfies Laplace's equation ∇^2^*c* = 0^1^,^2^,^3^,^27^,^28^,^29^. Hu and Larson^18^ showed that the heat transfer in drying droplets can also be considered as quasi-steady and the rate of the convective heat transfer is much smaller than that of the conductive one in the droplets. Ristenpart et al.^20^ further indicated that, although the liquid velocity diverges in the vicinity of the contact line, heat conduction is nonetheless dominant in the whole droplet. Therefore, inside the slowly evaporating droplets, the temperature field can be governed by Laplace's equation ∇^2^*T* = 0^18^,^20^,^23^,^24^. At the liquid-vapor interface, the vapor concentration *c* is assumed to be the saturation concentration which is assumed to be a linear increasing function of the local liquid temperature, namely,
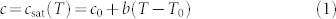
where *T* is the local liquid temperature at the droplet surface, 
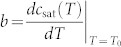
, and *c*_0_ = *c*_sat_(*T*_0_) is the saturated vapor concentration of the liquid at temperature *T*_0_^28^,^29^,^34^,^42^. *c*_∞_ = *Hc*_0_ is the vapor concentration far above the droplet, where *H* is the relative humidity of the ambient air. On the dry part of the substrate the mass flux is zero, i.e., ∂*c*/∂*z* = 0. Assuming that the heat conduction and convection in the air can be neglected^18^,^23^,^24^,^34^,^43^, the local energy balance on the liquid-vapor interface is
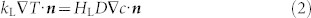
where *k*_L_ is the thermal conductivity of the liquid, *H*_L_ is the latent heat of evaporation, *D* is the diffusion coefficient of liquid vapor in the atmosphere, and ***n*** is the unit normal.

### Combined field approach

During drying, the heat conduction field inside the liquid droplet and the vapor diffusion field in the surrounding atmosphere interact with each other through the liquid-vapor interface: the diffusion-controlled evaporation will lower the temperature at the droplet surface, which can inversely affect the evaporative rate through its control on the saturation concentration of vapor right at the surface^18^,^27^,^28^,^29^. Thus, the liquid evaporation is a two-way coupling problem and numerical approaches have to be used to obtain the evaporation rate from the drying droplets.

Assuming that the vapor concentration at the droplet surface equals to a constant *c*_0_ (i.e., assuming that *b* = 0), Hu and Larson^27^ decoupled the vapor concentration in the atmosphere from the temperature in the droplet and numerically derived a simple approximate evaporation flux expression, namely,
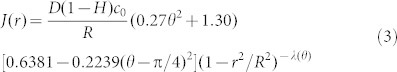
where *λ*(*θ*) * = * (1/2-*θ*/*π*). Although the analytical solution compares well with previous literature data, it does not account for the effect of the evaporative cooling. To obtain a more accurate result for the evaporation flux along the droplet surface, the temperature dependence of the saturation concentration of vapor must be included in the model. As a result, the numerical procedure is complex because iteration between the vapor diffusion field in the air and the heat conduction field in the droplet has to be adopted^28^,^29^,^30^,^31^.

From the above equations, it can be seen that both the two coupled fields in the liquid evaporation are governed by the Laplace's equation. This enables the possibility of unifying these two fields into one single physics field which is also governed by the same differential equation. All we need to do is transfer the boundary conditions at the interface between the coupled fields (e.g., equations 1–2 in the text) to the boundary conditions at the interface between two media in one single field. This may be achieved through the nondimensionalization of the physical quantities of the fields.

Here, by choosing the scaling factors for the nondimensionalization of the temperature in the liquid droplet and that of the vapor concentration in the air as follows: 
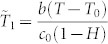
, and 
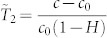
, the Laplace's equations and the boundary conditions in the droplet evaporation can be rewritten as
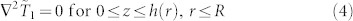

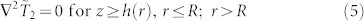

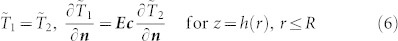

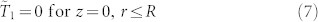

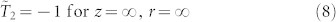

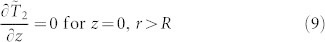
where *h*(*r*) is the height of the droplet, and 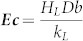 is a dimensionless number.

If we consider 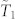, 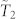 as the temperature in the liquid domain and that in the air domain respectively, equations (4–9) just represent the heat conduction in both the droplet region with thermal conductivity of 1 and the air region with thermal conductivity of ***Ec***. Thus, the coupled two fields have been unified into one single heat conduction field, and consequently, 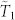 and 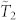 can be numerically solved from equations (4–9) without any iteration between the different fields. Once 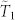 and 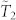 are known, the temperature in the droplet, the vapor concentration in the atmosphere, and the evaporation flux from the droplet surface can be easily computed.

### Evaporative cooling number *Ec*

It can be seen that, for a droplet with a given geometry, the above nondimensional equations for the droplet evaporation are governed only by the dimensionless number ***Ec***. Its definition implies that the number ***Ec*** combines the effects of the thermal properties of the liquid, the diffusion coefficient of liquid vapor in the atmosphere, and the temperature dependence of the saturation concentration of vapor.

For the case ***Ec*** = 0, 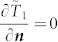 at the droplet surface (see equation 6). Considering also that 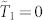 at the solid-liquid interface and 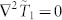 inside the droplet, 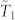 must be zero throughout the droplet, which means that the temperature of the whole droplet equals to the room temperature *T*_0_. So that the vapor concentration at the droplet surface must equal to the constant *c*_0_ and the problem for the vapor concentration in the atmosphere is decoupled from the problem for the temperature in the droplet. Thus, when ***Ec*** = 0, the present model reduces to the basic model developed by Picknett and Bexon^26^, Deegan et al.^2^, Popov^15^, and Hu and Larson^27^ in which the effect of evaporative cooling is neglected.

When ***Ec*** ≠ 0, a decrease in the surface temperature 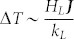 should be generated supposing that the local evaporation flux equals to ***J***, which in turn results in a decrease in the vapor concentration 
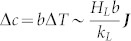
 at the droplet surface. This further implies a decrease in the evaporation flux from the droplet surface which can be approximately expressed as 
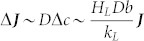
.

The above analysis shows that the evaporative cooling of the liquid will result in a decrease in the evaporation flux from the droplet surface. The intensity of the effect of the evaporative cooling on the droplet evaporation can be estimated by the ratio of the reduction in the evaporation flux Δ***J*** to its initial value ***J***, i.e., by the dimensionless number 
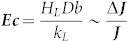
. The larger the value of ***Ec***, the more significant the negative feedback effect of evaporative cooling which reduces the evaporation rate: as ***Ec*** increases, it becomes progressively harder and harder to evaporate more quickly.

From its definition, it can be seen that the value of the number ***Ec*** is determined by the thermal properties of the liquid and the atmosphere. Under a temperature of 295 K and an atmospheric pressure of 99.8 kPa, ***Ec*** are 0.11, 0.84 and 1.03 for water, methanol and acetone in the air, respectively, and are 0.37, 3.34 and 4.13 for water, methanol and acetone in the helium, respectively^28^,^29^. Under the same conditions, methanol has a larger value of ***Ec*** than water. This may be one of the reasons why methanol has a more significant cooling effect when it evaporates from the skin.

### Influence of evaporative cooling on evaporation rate

**Figure 2:**
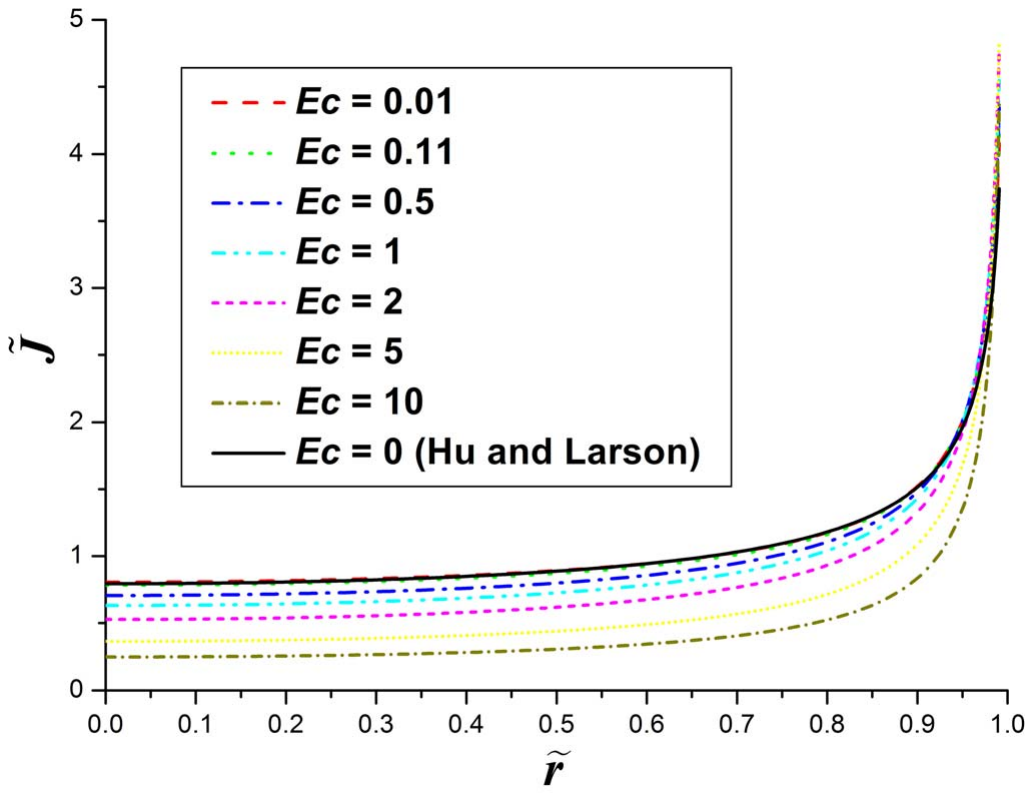
The nondimensional evaporation flux along the surface of drying droplets with the contact angle of *θ* = 20°. The solid line in the figure shows the solution obtained by Hu and Larson (equation 3 in the text).

To check the influence of the evaporative cooling on the liquid evaporation, the nondimensional evaporation flux 
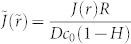
, where 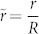
and *J*(*r*) = −*D*∇*c* · ***n*** is the evaporation flux from the droplet surface, as a function of 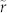 is calculated and then plotted in Figure 2. From the figure it can be seen that, as indicated above, the evaporation flux along the droplet surface decreases as the value of ***Ec*** increases. When ***Ec*** is small, the evaporation flux along the droplet surface is almost the same as in the case that the evaporative cooling effect is neglected (i.e., ***Ec*** = 0). The result shows that, for water droplets drying under an atmospheric pressure (***Ec*** ≈ 0.11^28^,^29^), the evaporative cooling has negligible influence on the evaporation rate. However, when ***Ec*** is larger than 1, the evaporation flux deviates significantly from the case without the evaporative cooling effect (i.e., the equation (3)) and can no longer be well fitted by the expression *J*_0_(*θ*)(1-*r*^2^/*R*^2^)^−*λ*(*θ*)^.

**Figure 3:**
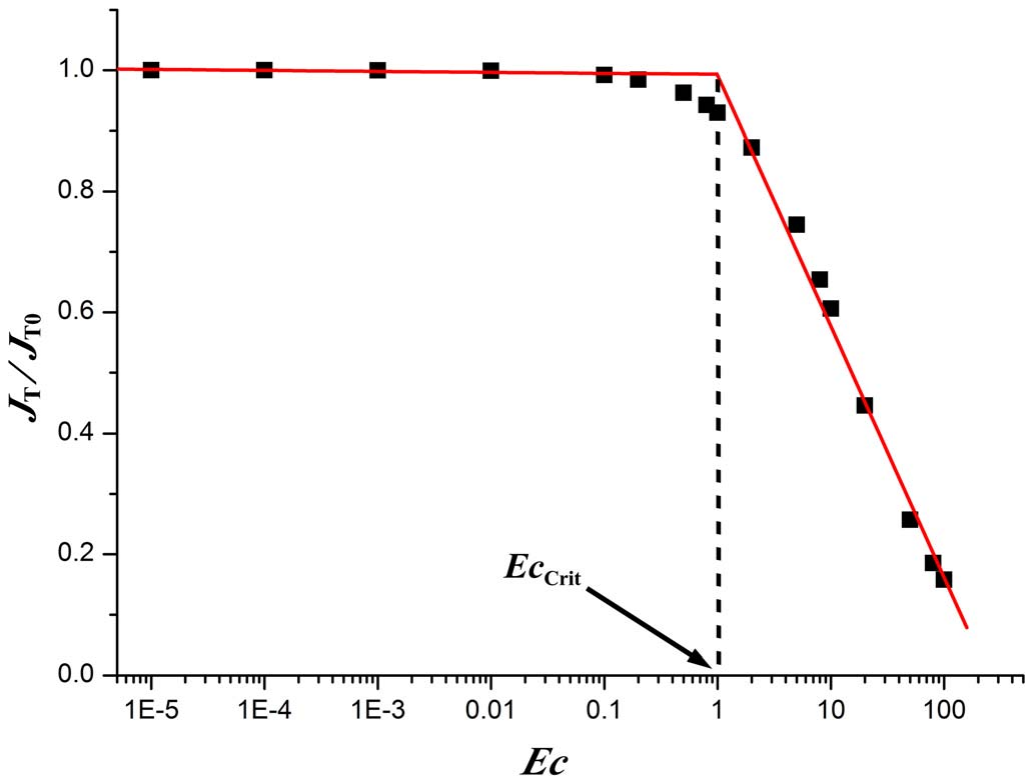
The total evaporation rate *J*_T_ from the drying droplets *vs* the log of *Ec*. *J*_T0_ is the total evaporation rate of the droplet when ***Ec*** = 0. The contact angle *θ* = 20°. The solid squares are from numerical calculations, and the two solid lines are the best linear fits to the data points within the range of ***Ec*** ≤ 0.1 and ***Ec*** ≥ 1 respectively (left line: *J*_T_/*J*_T0_ = 0.99327–0.00166 log*Ec*, the standard deviation of the fit is 0.00258; right line *J*_T_/*J*_T0_ = 0.99142–0.41493 log*Ec*, the standard deviation of the fit is 0.03629). The critical evaporative cooling number ***Ec*_Crit_** ≈ 1.

The variation of the total evaporation rate *J*_T_ from the droplet surface with ***Ec*** shown in the Figure 3 demonstrates more clearly the influence of the evaporative cooling on the droplet evaporation. As the ***Ec*** increases, the total evaporation rate decreases, which means that the evaporative cooling effect becomes more significant. From the figure it can be obviously seen that the variation of *J*_T_ can be divided into two regions, divided by a critical evaporative cooling number ***Ec*_Crit_**. When the evaporative cooling number is below the critical value, ***Ec*_Crit_**, the evaporation rate changes slightly as ***Ec*** changes, implying that the evaporative cooling has negligible influences on the droplet evaporation. When above ***Ec*_Crit_**, the evaporation rate decreases sharply with increasing ***Ec***. This means that the evaporative cooling effect should be taken into account when the evaporative cooling number ***Ec*** is above the critical value.

### Influence of evaporative cooling on surface temperature

**Figure 4:**
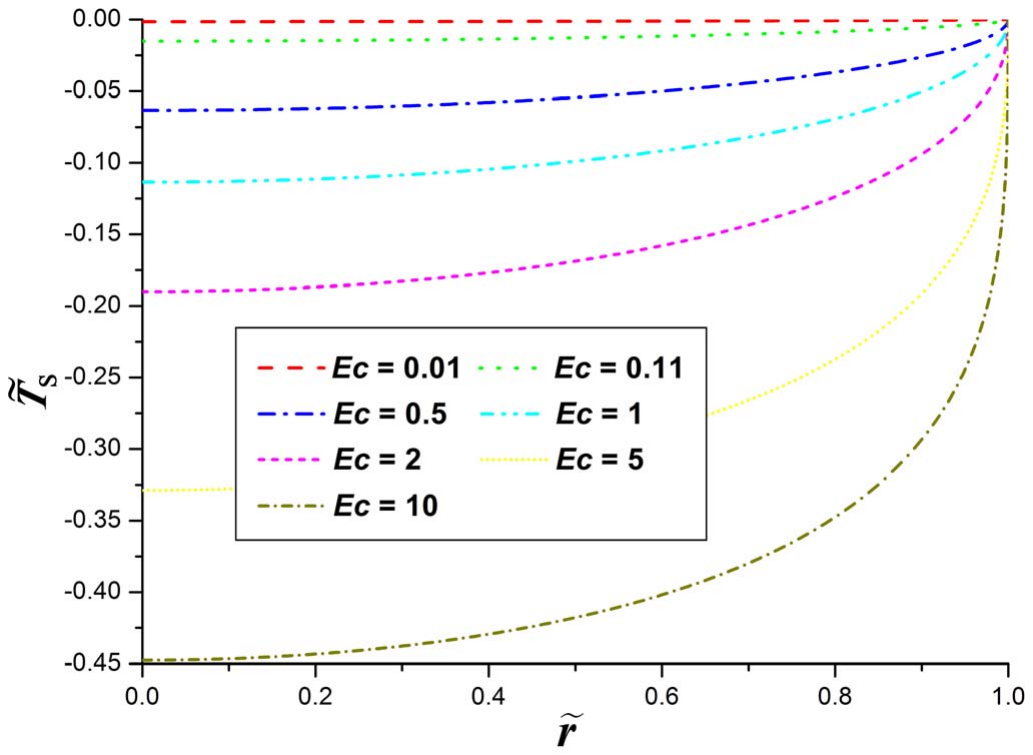
The nondimensional temperature along the surface of evaporating liquid droplets with the contact angle of *θ* = 20°.

To show the effects of the evaporative cooling on the thermal field of the droplet, the nondimensional temperature 
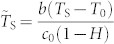
, where *T*_S_ is the liquid temperature at the droplet surface, is calculated and then plotted as a function of 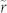 in Figure 4. From the figure it can be seen that the temperature increases monotonically along the droplet surface from the droplet center to the droplet edge, and finally reaches the room temperature at the contact line. The behavior is similar for all values of ***Ec***, and is consistent with previous results for drying droplets on isothermal surfaces^18^,^20^,^24^,^34^. This means that, on isothermal substrates, the evaporative cooling will not alter the trend of the temperature distribution along the droplet surface.

The figure also demonstrates the significant influence of the evaporative cooling on the surface temperature of drying droplets: the depression of the surface temperature 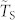 increases as the evaporative cooling number ***Ec*** increases. When ***Ec*** is close to zero, the nondimensional temperature 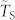 is also approaching zero over the whole droplet surface, and we can further deduce that the nondimensional temperature 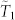 approaches zero throughout the droplet. In such a situation, the temperature throughout the whole droplet is approximately constant at the room temperature and then no evaporative cooling happens.

As the value of ***Ec*** increases, the evaporation-induced cooling along the droplet surface also becomes more significant. It can be seen from the Figure 4 that, when ***Ec*** is larger than 1, the nondimensional temperature 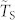 along the droplet surface deviates significantly from the case without the evaporative cooling effect (i.e., the case that the temperature throughout the droplet and thus over the droplet surface is constant at the room temperature).

### Comparisons with the experimental results

The agreement with the solution obtained by Hu and Larson^27^ can prove the validity of the approach in one limiting case. Here, to further verify the validity of the present model, comparisons with the previous experiments by Dunn et al.^28^,^29^, Sefiane et al.^21^,^30^, and Deegan et al.^2^ are performed.

**Figure 5:**
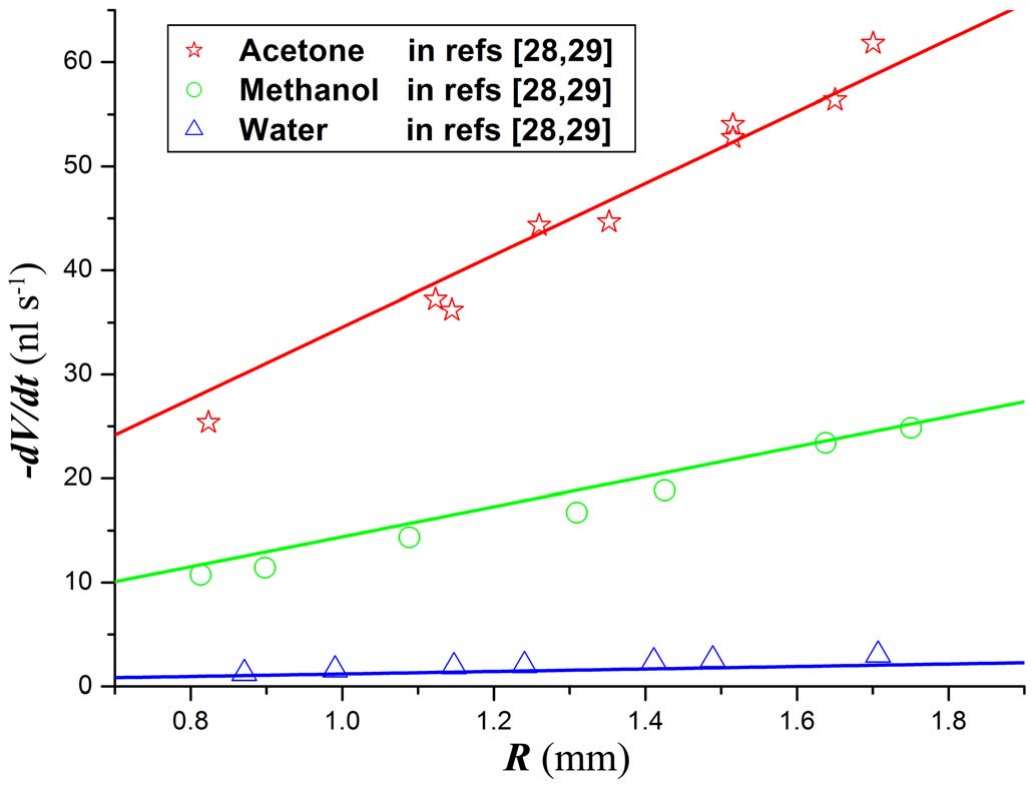
The total evaporation rate for droplets of three liquids as a function of the droplet base radius. The solid lines are from the present model (eqs 10 and 11). The ambient gas is air with fixed temperature 295 K, pressure 998 mbar, and relative humidity 0.4 for water and 0 for acetone and methanol. The results of aluminum substrate are used here because it can be approximately considered as isothermal substrate due to the high thermal conductivity of aluminum.

The total evaporation rate from the whole droplet surface can be expressed as
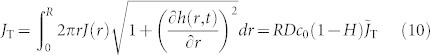
with the nondimensional total evaporation rate 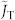 given by
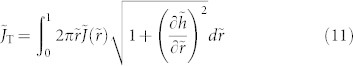
It can be deduced from above analyses that, for the diffusion-limited evaporation, 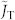 is just a function of *θ* and ***Ec***. Thus, the total evaporation rate of droplets is proportional to the droplet base radius (eq. 10), which is consistent with the experimental measurements by many previous authors, including Dunn et al., Sefiane et al. and Deegan et al. The comparison between the experimentally measured values for the total evaporation rate of droplets^28^,^29^ and the theoretical predictions of the present model (eqs 10 and 11) shows good agreement (see Fig. 5).

**Figure 6:**
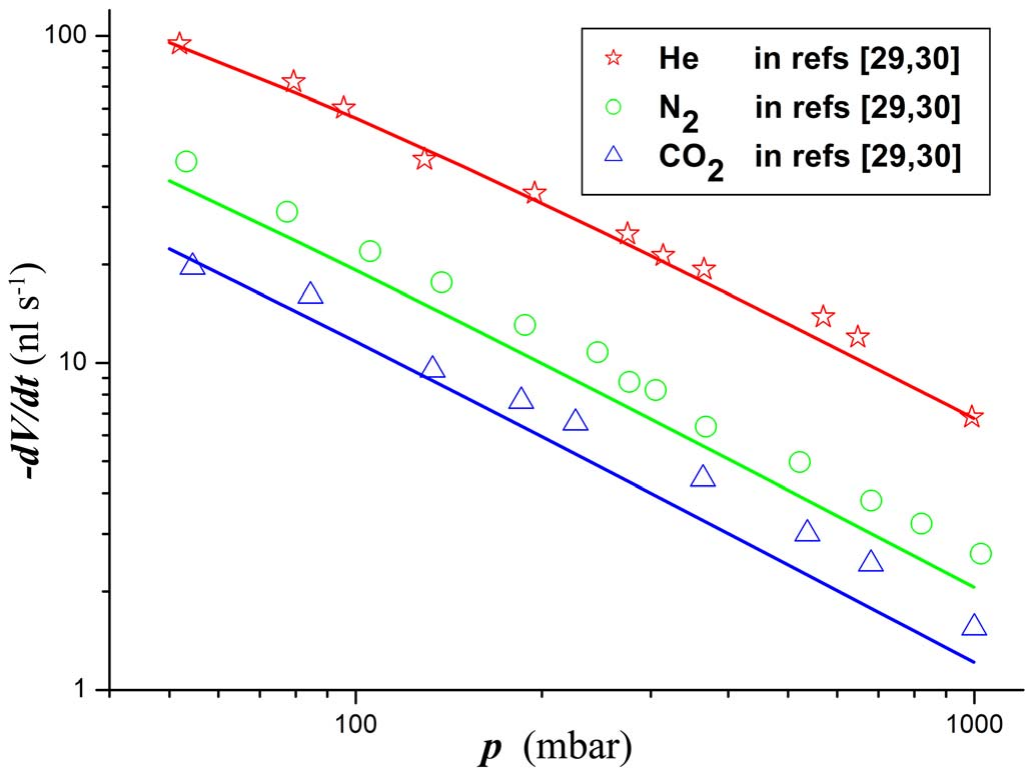
The total evaporation rate of water droplets on aluminum substrates in atmospheres of helium, nitrogen and carbon dioxide as a function of the atmospheric pressure. The solid lines are from the present model (eqs 10 and 11). The room temperature is 295 K and the relative humidity is 0.

Reducing the atmospheric pressure increases the diffusion coefficient of liquid vapor in the atmosphere, and thus increases the evaporation rate of droplets. To investigate the effect of the atmosphere and its pressure on the droplet evaporation, the total evaporation rate of water droplets in different gases with varied pressure is calculated from eqs 10 and 11. Fig. 6 shows that the theoretical predictions of the present model are in good quantitative agreement with the experimental results of Dunn et al.^29^ and Sefiane et al.^30^ The consistence shows that the present model is accurate for ***Ec*** as high as 7.42 (water droplet in helium with a pressure of 50 mbar).

Both the theoretical solutions and the measured data shown in Fig. 6 can be reasonably approximated by a straight line on the logarithmic scale. This leads to a numerical fit in the form −*dV*/*dt* ~ *p^β^*, where *β* is fitting parameter. When there is no evaporative cooling (i.e., ***Ec*** = 0), the total evaporation rate of droplets should be strictly inversely proportional to the atmospheric pressure *p* (i.e., *β* = −1*)* because *D* is inversely proportional to *p*^29^,^30^. However, when considering the evaporative cooling effect, *β* should be a value more or less larger than −1 because the increase in the diffusion coefficient *D* also results in an increase in ***Ec***, which in turn induces a reduction in the evaporation rate (Fig. 3). Fits of the present theoretical predictions yield *β* ≈ −0.898 for helium, *β* ≈ −0.962 for nitrogen, and *β* ≈ −0.976 for carbon dioxide, which are found to be in encouraging agreement with the experimental results obtained by Sefiane et al.^30^.

## Conclusions

By introducing an approach which combines the coupled field of temperature inside and vapor concentration outside the drying droplet into one “quasi-temperature” field and thus makes the numerical iteration between the couple fields unnecessary, we have characterized the effect of evaporative cooling during droplet evaporation. The agreement with the limiting solution obtained by Hu and Larson^27^ and with the experimental data measured by Dunn et al.^28^,^29^ and Sefiane et al.^21^,^30^ proved the validity of the approach, which may also be applicable to other problems in which coupled fields have the same governing equation.

By using the combined field approach, a dimensionless number ***Ec*** was derived to evaluate the strength of the evaporative cooling effect during the liquid evaporation. When the contact line of droplets is pinned (i.e., constant contact radius mode^44^), both the evaporation flux along the droplet surface and the total evaporation rate of the droplet will decrease as ***Ec*** increases. A critical value ***Ec*_Crit_** exists below which the evaporative cooling effect can be neglected and above which the significance of the effect increases dramatically. For the cases of other evaporation modes (e.g., constant contact angle mode, stick–slide mode^44^), the present approach is also applicable provided that the evaporation is diffusion-limited.

Because of the use of the linear dependence of the saturation concentration of vapor on the temperature, the theory presented here is appropriate for the slowly evaporating droplets where the evaporative cooling is relatively small. But, it can be used as a framework for addressing the more complicated situations in which a large evaporative cooling occurs and thus more realistic expressions for the saturation concentration of vapor with temperature (e.g., the quartic approximation^30^) should be used. Despite its simple origin and limitations, the results presented here may serve as an attempt to understand thoroughly the evaporative cooling effect in the liquid evaporation and to investigate its influences on the evaporation of pinned sessile droplets, and thus may be useful to predict and control the flow field and the deposition pattern of drying droplets.

## Author Contributions

X.X. and L.M. wrote the main manuscript text, and X.X. prepared the figures. All authors reviewed the manuscript.
